# Cyanoacrylate Tissue Adhesives Compared With Sutures on Facial and Neck Wounds: A Meta‐analysis

**DOI:** 10.1002/oto2.73

**Published:** 2023-09-08

**Authors:** Prapitphan Charoenlux, Nattawan Utoomprurkporn, Kachorn Seresirikachorn

**Affiliations:** ^1^ Department of Otolaryngology Faculty of Medicine, Chulalongkorn University Bangkok Thailand; ^2^ Faculty of Brain Science, UCL Ear Institute University College London London UK; ^3^ Endoscopic Nasal and Sinus Surgery Excellence Center King Chulalongkorn Memorial Hospital Bangkok Thailand

**Keywords:** cyanoacrylate tissue adhesives, facial and neck wound, suture

## Abstract

**Objective:**

To compare the effectiveness between cyanoacrylate tissue adhesives (CTAs) and sutures for skin closure on the face and neck.

**Data Sources:**

Embase, Medline, Scopus, Central, Web of Science.

**Review Methods:**

Randomized controlled trials comparing CTAs versus sutures for skin closure on the face and neck were included. Primary outcomes were cosmetic outcomes. Secondary outcomes were scar depth, scar width, pain, closure time, cost, and adverse events. Subgroup analyses were performed by wound locations, type of CTAs, type of sutures, age groups, and type of wounds. Physicians and patients evaluated the cosmetic outcomes.

**Results:**

Eighteen studies (1020 patients) were included. CTAs offered better cosmetic outcomes by Wound Registry Scale at ≤1 month (physician: mean difference [MD]: −1.50, 95% confidence interval, CI: −2.42 to −0.58). The cosmetic outcomes assessed by Visual Analog Scale were comparable at >1 to ≤3 months (physicians: standard mean difference [SMD], −0.01, 95% CI, −0.25 to 0.23, patients: SMD, −0.02, 95% CI, −0.84 to 0.79). The cosmetic outcomes by the Patient and Observer Scar Assessment Scale favored sutures at >3 to 12 months (physician: MD 4.26, 95% CI, 2.02‐6.50). Subgroup analyses revealed no differences. CTAs offered less scar depth, scar width, pain, closure time, and total cost of closure. Adverse events were similar.

**Conclusion:**

Based on the wound healing process, the cosmetic outcomes exhibited a favorable inclination toward CTAs at <1 month while demonstrating comparable results between CTAs and sutures at >1 to ≤3 months. Subsequently, sutures exhibited superior cosmetic outcomes compared to CTAs at >3 to 12 months.

Tissue adhesive is classified into 3 categories: natural, synthetic/semisynthetic, and biomimetic subtypes.^1^, ^2^ Cyanoacrylate‐based adhesives are the most common material used in the synthetic group and have been used since the 1950s.^3^, ^4^ The properties of cyanoacrylate tissue adhesives (CTAs) are determined by the molecular structure of the alkyl side chains. CTAs can be classified into 2 categories (5 groups)^5^: (1) short‐chain CTAs (methyl‐2‐cyanoacrylate and ethyl‐2‐cyanoacrylate)^5^ and (2) long‐chain CTAs (n‐buthyl‐2‐cyanoacrylate, isobutyl‐cyanoacrylate, and 2‐octyl‐cyanoacrylate).^5^ Although short‐chain CTAs form tighter and stronger bonds than long‐chain CTAs, the bonds are fragile and easily fractured, resulting in a low tensile strength.^6^ In addition, short‐chain cyanoacrylates can cause tissue toxicity due to their alkyl side chains which rapidly degrade into cyanoacetate and formaldehyde and result in local tissue accumulation and inflammation.^7^ The use of long‐chain CTAs has increased due to their longer degradation durations which can decrease histotoxicity.^5^, ^6^, ^7^ After polymerization, a CTA forms a plastic film to approximate the wound edges.^8^ Typically, the film sloughs off in 5 to 10 days.^9^ Its tensile strength was similar to a 5‐0 nonabsorbable suture material.^8^ Therefore, CTAs are suitable for low‐tension, laceration, or surgical incision wounds.^8^, ^10^

Even though suturing is the most common technique for skin closure, there are some potential drawbacks, such as a possible requirement of local anesthetic injection, additional cost of wound dressing and local antimicrobial medication, difficult application in children, and needle‐stick injury.^9^, ^11^ In contrast, the advantages of CTAs are fast, simplicity of application, and no stitch removal. However, their use is limited to only specific wound characteristics such as laceration wounds. Moreover, there are some drawbacks, such as tissue toxicity from monomers or degradation products, including formaldehyde, cyanoacetate, and exothermal reaction.^12^ Although, CTAs are widely employed as a suture alternative in various operations,^13^, ^14^, ^15^, ^16^ including skin closure of the facial and neck wounds. There is a lack of strong evidence comparing the effectiveness of the CTAs versus sutures, especially on facial and neck wounds which are exposure areas and highly cosmetic concern locations.^17^ Some reports showed a better cosmetic score on sutures, while others showed a comparable outcome.^18^, ^19^, ^20^, ^21^ Moreover, most of these studies focused only on the long‐term results. The wound locations and closure methods (staple, sterile strip, suture) that the studies used to compare with CTAs were mixed.^18^, ^19^, ^20^, ^21^ This systematic review and Meta‐analysis aimed to compare the effectiveness regarding cosmetic outcomes, scar depth, scar width, pain, closure time, cost, and adverse events, between CTAs and sutures for skin closure of facial and neck wounds.

## Methods

This systematic review was carried out following the Preferred Reporting Items for Systematic Reviews and Meta‐analyses statement.^22^ This protocol was registered with PROSPERO (CRD42021258608). This study was waived from a review by the Institutional Review Board of the human or animal study because the study examined only the data from published literature.

### Information Sources and Search Strategy

A systematic search was performed on the Ovid Embase, Ovid Medline, Scopus, Central, and the Web of Science, without any restriction on the year of publication. The search terms included: “tissue glue OR tissue adhesive OR adhesive glue OR cyanoacrylate” AND “suture or sutur*” AND “facial OR face OR neck.” References of the included studies were searched to identify any missing published or unpublished trials. The last updated search was conducted on December 12, 2022.

### Eligibility Criteria

Randomized controlled trials (RCTs) on humans that compared CTAs versus sutures for skin closure of laceration or surgical incision wounds on the face or neck were included. There were no limitations on age, type of CTAs, type of sutures, or treatment duration. The RCTs with the following wound types: (1) puncture wound, (2) animal or human bite wound, (3) ulcerative decubitus wound, (4) crush wound, (5) contaminated/infected/or devitalized wound, and (6) mucosal or mucocutaneous junction wound were excluded. Reviews, meeting abstracts, comments, and studies published in languages other than English were excluded.

### Study Selection and Data Collection

Two reviewers (P.C., K.S.) independently screened the titles and abstracts based on the predetermined eligibility criteria. The full texts of the selected articles were reviewed. When there was insufficient information for data extraction or imputation, the corresponding authors of the studies were contacted for more information. Any conflict during screening and data extraction was resolved by discussions among the authors or the final decision by the third author (N.U.). Data were extracted independently by 2 reviewers (P.C., K.S.) using a predetermined data collection form. The extracted data included the first author, year of publication, number of participants, age, wound characteristics, cyanoacrylate type, suture type, size of suture material, closure method, primary outcomes, and secondary outcomes. If more than 1 type of suture material was used in the trial, the more commonly used material was selected in the suture group. Primary outcomes were cosmesis assessed by the Wound Registry Scale, scar quality score, Visual Analog Scale (VAS), and the Patient and Observer Scar Assessment Scale (POSAS). If multiple tools were used in the cosmetic assessments in each analysis period, the tool used by the most included articles was selected for the Meta‐analysis. If the cosmetic evaluation tools were equally used by the included articles in each analysis period, all the analysis tools were selected for the Meta‐analysis. Secondary outcomes were scar depth, scar width, pain evaluated by the VAS, closure time, cost, and adverse events.

### Risk of Bias Assessment

The risk of bias in each study was independently assessed following the *Cochrane Handbook for Systematic Reviews of Interventions*.^23^ by 2 reviewers (P.C., K.S.). Any discrepancy was resolved by discussion between the reviewers. The risk of bias was evaluated in 6 domains which included randomization, allocation concealment, blinding of participants and personnel, blinding of outcome assessment, incomplete outcome data, and selective outcome reporting. Each domain was determined as low risk, high risk, or unclear, based on the Cochrane risk of bias assessment criteria.^24^, ^25^

### Data Synthesis and Statistical Analysis

Data were pooled for the Meta‐analysis. Descriptive statistics were used to describe the study population characteristics. Mean difference (MD), standard mean difference (SMD), and 95% confidence interval (CI) were used for continuous data, such as cosmetic outcomes, pain, and closure time. The risk ratio (RR) and 95% CI of adverse events were calculated for dichotomous data and used for pooled comparisons. Heterogeneity or discrepancy in the treatment effect estimation from different trials was assessed by an *I* ^2^ statistic which is used to indicate the level of heterogeneity. An *I* ^2^ of less than 40%, 40% to 60%, or more than 60% represented low, moderate, or substantial heterogeneity, respectively. A fixed‐effect model was adopted when the heterogeneity was low. A random‐effects model was used to estimate the difference when the heterogeneity was high. Statistical calculations were performed using Microsoft Excel and Review Manager (RevMan) version 5.4.^26^ Cosmetic outcomes were assessed with (1) the VAS, the score ranged from zero (the worst) to 10 (cm) or 100 (mm) (the best)^27^, ^28^; (2) the wound registry scale, the score ranged from zero (the worst) to 5 (the best)^29^; (3) the POSAS which included 2 subscales: the Patient Scar Assessment Scale (PSAS) which assessing on 6 categories including pain, itchiness, color, stiffness, thickness, and regular skin, and the Observer Scar Assessment Scale which assessing on 6 categories including vascularization, pigmentation, thickness, relief, pliability, and surface area. The score in each category ranges from 1 (minimum) to 10 (maximum), so the summed score of 6 was the best possible scar and the worst possible scar was 60 in each subscale^30^, ^31^, ^32^; and (4) the scar quality score, ranging from 1 (excellent) to 5 (poor).^33^ The measurement tools were validated as a valid scale for assessing cosmetic scars. The time points for analysis were based on the wound healing phase as follows^34^, ^35^: (1) ≤1 month: inflammatory and proliferative phase, (2) >1 to ≤3 months: early remodeling phase, and (3) >3 to 12 months: late remodeling phase. Subgroup analyses were performed by wound locations (face, neck, face, and neck), age groups (children ≤18 years old, adults >18 years old), type of CTAs (short‐chain, long‐chain), type of sutures (absorbable, nonabsorbable), and type of wounds (surgical incision, laceration). Sensitivity analysis was performed by excluding the highest extreme values of the enrolled studies. Publication bias was evaluated using funnel plots. Statistical significance was defined when a *P* value was less than .05.

## Results

### Study Selection

**Figure 1:**
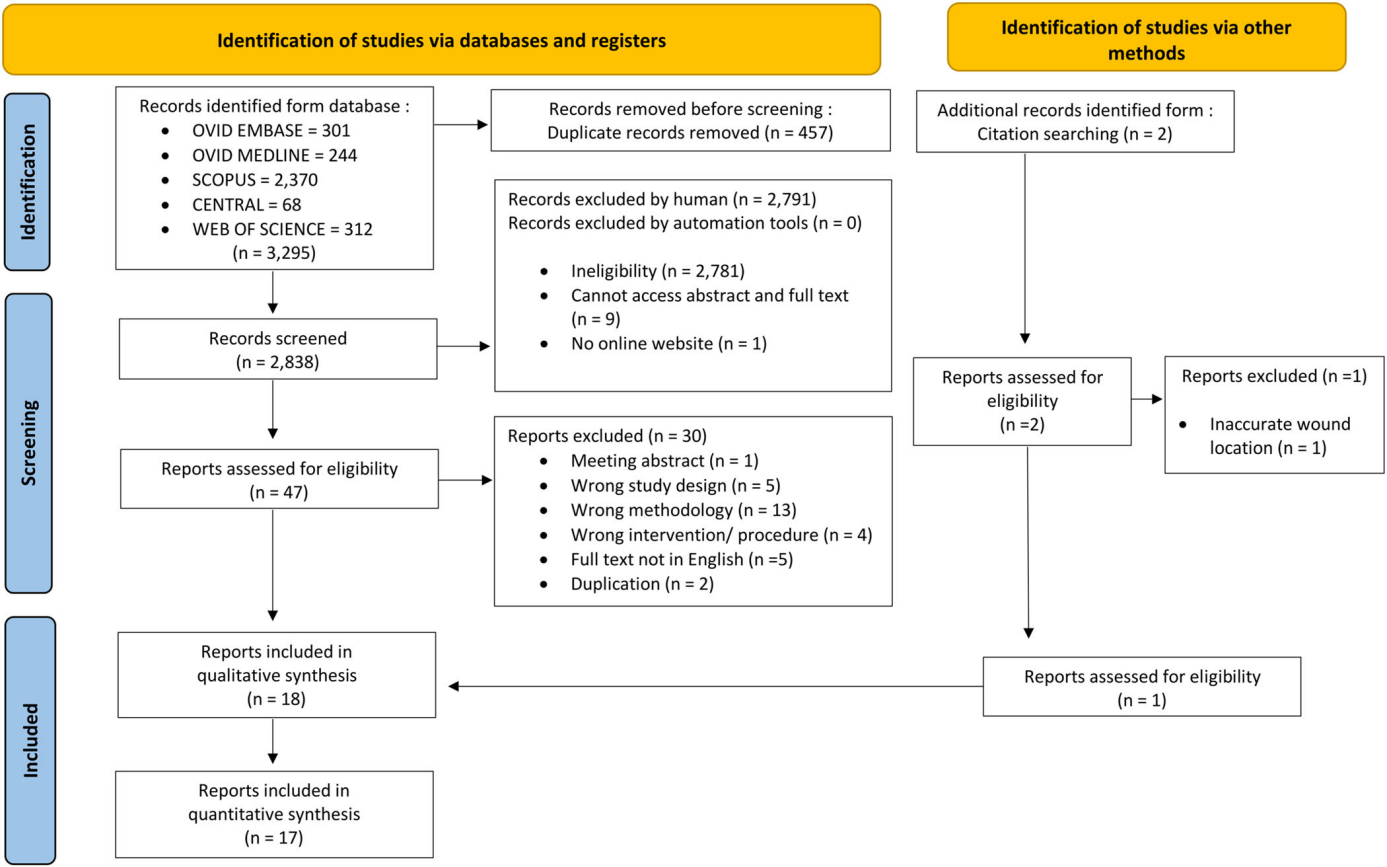
A flowchart of the study retrieval and selection: The Preferred Reporting Items for Systematic Reviews and Meta‐analyses flowchart of the systematic literature reviews.

There were 3297 studies initially identified and retrieved, of which 3295 were from the database and register search, and 2 were from citation search. After 457 duplications were removed, 2838 records remained for title and abstract screening, and 2791 records were excluded due to irrelevant references. Finally, the full text of 49 studies was reviewed. Eighteen articles^33^, ^36^, ^37^, ^38^, ^39^, ^40^, ^41^, ^42^, ^43^, ^44^, ^45^, ^46^, ^47^, ^48^, ^49^, ^50^, ^51^, ^52^ were included in the qualitative synthesis, of which 17^33^, ^36^, ^37^, ^38^, ^39^, ^40^, ^41^, ^42^, ^43^, ^44^, ^45^, ^46^, ^47^, ^49^, ^50^, ^51^, ^52^ were included in the quantitative synthesis. Figure 1 shows the flowchart of study retrieval and selection.^22^

### Study Characteristics

The publication year of the included articles ranged from 1993 to 2021. Characteristics of the included studies were presented in Table 1 and the characteristics of outcome assessments were presented in Table 2.

**Table 1:** The Characteristics of the Included Studies

Reference	Targeted population^a^	N (pt)	Age, y (mean ± SD)	Wound (location/type)	Intervention arm		Control arm			
					Type of cyanoacrylate	N (pt)	Type and size of suture	Absorbable/nonabsorbable	Methods of closure	N (pt)
Quinn et al^36^	Children	75	N/A	Face/laceration	N‐2‐butylcyanoacrylate (Histoacryl Blue)	37	Monofilament 5‐0, 6‐0	Absorbable	N/A	38
Greene et al^37^	N/A	20^b^	N/A	Face/surgical incision	Octyl‐2 cyanoacrylate	20^b^	Gut 6‐0	Absorbable	Running	10^b^
							Polypropylene 6‐0	Nonabsorbable	N/A	10^b^
Ozturan et al^38^	N/A	101	N/A	Face/surgical incision	Butylcyanoacrylate (Liquiband)	34	Polypropylene 6‐0	Nonabsorbable	Interrupted	67
Holger et al^39^	Children	84	N/A	Face/laceration	Octylcyanoacrylate	27	Nylon 6‐0	Nonabsorbable	N/A	28
							Gut 6‐0	Absorbable	N/A	29
Handschel et al^40^	N/A	45	N/A	Face/surgical incision	Octyl‐2 cyanoacrylate (Dermabond)	19	Ethilon (monofilament) 6‐0	Nonabsorbable	N/A	26
Sniezek et al^41^	N/A	14^b^	72 ± 8.8	Face&neck^#^/surgical incision	Octyl‐2 cyanoacrylate (Dermabond)	14^b^	Polypropylene 5‐0	Nonabsorbable	Interrupted	14^b^
Shivamurthy et al^42^	N/A	19	N/A	Face&neck/N/A	Octyl‐2 cyanoacrylate (Dermabond)	9	Silk (size N/A)	Nonabsorbable	N/A	10
Kouba et al^33^	N/A	36	55.8 ± 5.4	Face/surgical incision	Ethyl‐cyanoacrylate	12	Gut (monofilament) 6‐0	Absorbable	N/A	12
							Polypropylene (monofilament) 6‐0	Nonabsorbable		12
Soni et al^44^	Adults	28	N/A	Face&neck/surgical incision	Octyl‐2 cyanoacrylate (Dermabond)	13	Polypropylene (monofilament) 6‐0	Nonabsorbable	N/A	15
Consorti et al^43^	N/A	50	N/A	Neck/surgical incision	Octyl‐ cyanoacrylate	25	Polyglacin 3‐0	Absorbable	N/A	25
Alicandri‐Ciufelli et al^45^	N/A	89	53.36 ± 14.18	Neck/surgical incision	Dermabond	42	N/A	N/A	N/A	47
Kim et al^46^	Adults	13^b^	67.5 ± 14.5	Face/surgical incision	n‐butyl and 2‐octyl cyanoacrylate (GluSeal)	13^b^	Gut 5‐0, 6‐0	Absorbable	Running	13^b^
Rao et al^47^	N/A	74	N/A	Neck/surgical incision	Octylcyanoacrylate (Marvilyte)	36	Ethilon 3‐0	Nonabsorbable	N/A	38
Sahu et al^49^	Adults	24	N/A	Face&neck/surgical incision	N‐buytl cyanoacrylate (REKSEAL)	12	Nylon 4‐0	Nonabsorbable	Simple interrupted	12
Teoh et al^50^	Adults	96	52 ± 13.8	Neck/surgical incision	Octyl‐2‐cyanoacrylate and n‐2‐butylcyanoacrylate (Leukosan)	49	Braided polyglycolic acid 4‐0	Absorbable	N/A	47
Dinakar et al^48^	N/A	84	N/A	Face/laceration	Octyl‐2 cyanoacrylate (Dermabond)	28	Nylon (monofilament) 8‐0	Nonabsorbable	Interrupted	28
							Nylon (monofilament) 5‐0	Nonabsorbable	N/A	28
Chung et al^51^	N/A	126	N/A	Neck/surgical incision	n‐butyl‐2‐cyanoacrylate (Leukosan)	42	N/A	Absorbable	N/A	42
					n‐butyl‐2‐cyanoacrylate (Leukosan) + adjunctive laser and steroid injection	42				
Kumar et al^52^	Adults	42	24.61 (SD N/A)	Neck/surgical incision	Cyanoacrylate (type N/A)	14	Nylon (monofilament) 4‐0	Nonabsorbable	Interrupted	14
							Staple			14

“Face&neck^#^” referred to wounds spanning both regions or the studies considered wounds in both areas without separate analysis

Abbreviations: N/A, not available from the original data sources; pt, patient(s); SD, standard deviation.

^a^ Targeted population: Adults: ≥18 years old, children: <18 years old.

^b^ The wound was divided into the control half and the experimental half.

**Table 2:** The characteristics of outcome assessments

Reference	Assessment			
	Timing of evaluation	Outcomes	Tools	Assessors
Quinn et al^36^	After procedure	Pain	VAS	Parent of the patient
		Time		Physician
	5 d	Infection, dehiscence		N/A
	3 mo	Cosmesis	VAS, categorical scale	Physician
Greene et al^37^	After procedure	Closure time, closure quality		Physician
	POD 1, 1 wk, 2 wk, 4 wk	Cosmesis	VAS, modified HWES	Physician, patient
Ozturan et al^38^	After procedure	Time, cost		N/A
	3 mo	Cosmesis	VAS	Physician
			HWES	Physician
		Infection, inflammation, dehiscence, scarring		N/A
Holger et al^39^	4‐5 d	Infection, dehiscence		Physician
	9‐12 mo	Cosmesis	VAS	Physician, patient
Handschel et al^40^	10 d	Infection, dehiscence		N/A
	3 mo	Cosmesis	VAS	Physician, patient
		Scar depth		Physician
Sniezek et al^41^	1 wk	Infection, Inflammation, Dehiscence		N/A
	3 mo	Cosmesis	VAS	Physician
Shivamurthy et al^42^	After procedure	Time		N/A
	10 d	Infection, dehiscence		N/A
	2 mo	Cosmesis	VAS	N/A
Kouba et al^33^	1 wk	Dehiscence, SE (itching, bleeding, pain)		N/A
	1, 3 mo	Patient preference		Patient
		Cosmesis	Scoring of scar quality	Physician
Soni et al^44^	After procedure	Time		N/A
	5‐10 d	Healing, infection, inflammatory reaction		N/A
	3 mo	Cosmesis appearance	Modified HWES	Physician
		Cosmesis scar	VAS	Physician
		Patient satisfaction	VAS	Patient
Consorti et al^43^	After procedure	Time		N/A
	6 wk	Cosmesis	POSAS (PSAS, OSAS)	Physician, patient
Alicandri‐Ciufelli et al^45^	10 d	Cosmesis	Wound Registry scale	Physician
	3 mo	Cosmesis	SBSES	Physician
Kim et al^46^	After procedure	Complication		N/A
	3 mo	Cosmesis	VAS	Physician
		Patient preference	Patient preference	Patient
Rao et al^47^	1, 3 wk	Cosmesis	SBSES	N/A
		Pain	VAS	N/A
Sahu et al^49^	After procedure	Time		N/A
	1, 3, 7 d	Dehiscence, necrosis, infection		N/A
Teoh et al^50^	7 d	Initial inspection		Physician
	6 wk	Cosmesis	SBSES	Physician
	3 mo	Cosmesis	POSAS	Physician
Dinakar et al^48^	After procedure	Time		N/A
	1, 7 d	Healing, infection, inflammation		N/A
	3 mo	Cosmesis	Modified HWES	Physician
Chung et al^51^	1, 2 mo	Cosmesis	No assessment	N/A
	6 mo	Cosmesis	POSAS	Physician, patient
		Scar width at midpoint and widest point		Physician
Kumar et al^52^	POD 7, 2 wk, 1 mo	Infection, inflammation, dehiscence		N/A
	3 mo	Cosmesis	Modified HWES, VAS	Physician, patient
	2, 3, 4 mo	Hypertrophic scar		N/A

Abbreviations: d, day; HWES, Hollander Wound Evaluation Scale; mo, month; N/A, not available from the original data sources; OSAS, Observer Scar Assessment Scale; POD, postop day; POSAS, Patient and Observer Scar Assessment Scale; PSAS, Patient Scar Assessment Scale; SBSES, Stony Brook Scar Evaluation Scale; SE, side effect; VAS, Visual Analog Scale.

### Participants

Among the 18 included studies,^33^, ^36^, ^37^, ^38^, ^39^, ^40^, ^41^, ^42^, ^43^, ^44^, ^45^, ^46^, ^47^, ^48^, ^49^, ^50^, ^51^, ^52^ 1020 patients were enrolled and randomized to receive a CTA or sutures for skin closure. The mean age was 60.13 ± 11.34 years, being 53.31% female. The wound types are presented in Table 1. The wounds were located on the face (8 studies),^33^, ^36^, ^37^, ^38^, ^39^, ^40^, ^46^, ^48^ the neck (6 studies),^43^, ^45^, ^47^, ^50^, ^51^, ^52^ and both the face and neck (4 studies).^41^, ^42^, ^44^, ^49^ In the CTA group, butylcyanoacrylate (n‐2‐butyl, butyl, n‐butyl) was applied in 4 articles,^36^, ^38^, ^49^, ^51^ octylcyanoacrylate (octyl‐2, octyl) in 10 articles,^37^, ^39^, ^40^, ^41^, ^42^, ^43^, ^44^, ^45^, ^47^, ^48^ ethylcyanoacrylate in 1 article,^33^ a combination of butylcyanoacrylate (N‐butyl, N‐butyl‐2), and octylcyanoacrylate (octyl‐2) in 2 articles.^46^, ^50^ The type of CTA was not mentioned in 1 article.^52^ In the suture group, absorbable materials were used in 5 studies,^36^, ^43^, ^46^, ^50^, ^51^ nonabsorbable materials in 9 studies,^38^, ^40^, ^41^, ^42^, ^44^, ^47^, ^48^, ^49^, ^52^ and both absorbable and nonabsorbable materials in 3 studies.^33^, ^37^, ^39^ One study^45^ did not provide suture material data.

### Intervention

After hemostasis was achieved, the deep layer (deep dermal, subcutaneous, or muscle) was closed, if necessary, to relieve tension, obliterate space, or aid in wound edge approximation.^33^, ^38^, ^39^, ^40^, ^41^, ^43^, ^44^, ^45^, ^46^, ^48^, ^49^, ^52^ In the CTA group, the adhesive ampule was gently crushed just before application. Then, the adhesive was expressed through the tip applicator and mixed with a chemical compound for polymerization. The wound edges were meticulously approximated. The adhesive was brushed over the wound surface and at 2 to 10 mm lateral to the wound edge of each side. Ten to 30 seconds were allowed for complete polymerization. Another 1 or 2 additional layers were applied 5 to 15 seconds apart. In the suture group, after the wound margin was held to aid in apposition and ensure adequate eversion, the skin was closed using interrupted^38^, ^41^, ^48^, ^49^, ^52^ or continuous^37^, ^46^ fashion with 3‐0,^43^, ^47^ 4‐0,^49^, ^50^, ^52^ 5‐0,^36^, ^41^, ^46^, ^48^ 6‐0,^33^, ^36^, ^37^, ^38^, ^39^, ^40^, ^44^, ^46^ or 8‐0^48^ sutures.

### Outcomes

#### Cosmetic Outcomes

The scars were evaluated at 3 different periods which included ≤1,^33^, ^37^, ^45^ >1 to ≤3,^36,38,41‐44,46,52^ and >3 to 12 months.^39^, ^51^ The cosmetic outcomes were evaluated at almost all the time points by both physicians and patients.

##### ≤1 Month

###### Wound Registry Scale

There was 1 study^45^ that analyzed cosmetic outcomes using the Wound Registry Scale. The result significantly favored the CTA group (physician: MD, −1.50, 95% CI, −2.42 to −0.58, *P* = 0.001, 1 RCT^45^).

###### Scar Quality

One study analyzed cosmetic outcomes using scar quality.^33^ There were no significant differences between both groups (physician: MD, −0.29, 95% CI, −0.69 to 0.11, *P* = 0.15, 1 RCT^33^; patient: MD, −0.00, 95% CI, −2.04 to 2.04, *P* = 1.00, 1 RCT^33^).

##### >1 to ≤3 Months

###### VAS

**Figure 2:**
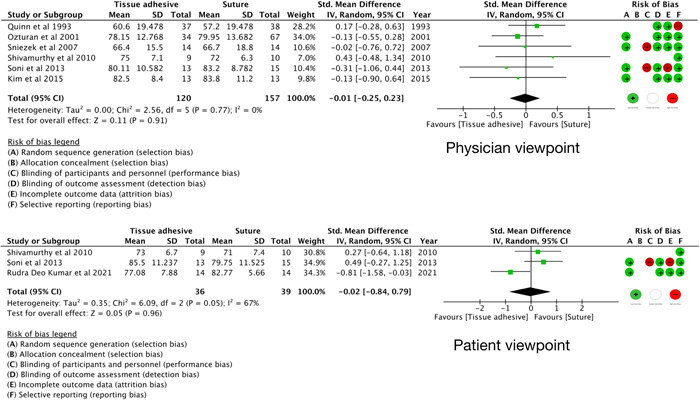
Cosmetic results from Visual Analog Scale at >1 to ≤3 months assessed by physicians and patients. CI, confidence interval; Fixed, fixed effect; IV, inverse variance; Random, random effects; Std., standardized mean difference.

Seven studies^36^, ^38^, ^41^, ^42^, ^44^, ^46^, ^52^ reported cosmetic results using the VAS. There were no significant differences between both groups (physician: SMD, −0.01, 95% CI, −0.25 to 0.23, *P* = 0.91, 6 RCTs,^36^, ^38^, ^41^, ^42^, ^44^, ^46^ *I* ^2^ = 0%; patient: SMD, −0.02, 95% CI, −0.84 to 0.79, *P* = 0.96, 3 RCT,^42^, ^44^, ^52^ *I* ^2^ = 67%) (Figure 2).

##### *>3* to 12 Months

###### POSAS

One study^51^ reported cosmetic results using the POSAS. The results favored sutures over CTAs. with statistical significance (physician: MD, 4.26, 95% CI, 2.02‐6.50, *P* = 0.0002, 1 RCT^51^; patient: MD, 4.29, 95% CI, 0.89‐7.69, *P* = 0.01, 1 RCT^51^).

###### VAS

One study^39^ reported cosmetic results using the VAS. There were no significant differences between both groups (physician: MD, −0.95, 95% CI, −10.36 to 8.46, *P* = 0.84, 1 RCT^39^; patient: MD, −1.20, 95% CI, −9.69 to 7.29, *P* = 0.78, 1 RCT^39^).

##### Subgroup Analysis

All subgroup analyses were analyzed using the VAS at >1 to ≤ 3 months. Subgroup analysis by wound location showed no significant differences between groups: the face (physician: SMD, −0.01, 95% CI, −0.30 to 0.27, *P* = 0.93, 3 RCTs,^36^, ^38^, ^46^ *I* ^2^ = 0%), and the face‐and‐neck (physician: SMD, −0.02, 95% CI −0.47 to 0.44, *P* = 0.95, 3 RCTs,^41^, ^42^, ^44^ *I* ^2^ = 0%; patient: SMD, 0.40, 95% CI, −0.18 to 0.98, *P* = 0.18, 2 RCTs,^42^, ^44^ *I* ^2^ = 0%). The results favored CTAs in the neck subgroup (patient: MD, −0.81, 95% CI, −1.58 to −0.03, *P* = 0.04, 1 RCT^52^). The subgroup difference was not significant in the physician's viewpoint (*P* = 0.99). The subgroup difference in patient viewpoint was significant (*P* = 0.01). There was no data on the neck subgroup in the physician aspect and the face subgroup in the patient aspect.

When subgroup analysis by suture type was performed, there were no significant differences between absorbable (SMD, 0.10, 95% CI, −0.30 to 0.49, *P* = 0.63, 2 RCTs,^36^, ^46^ *I* ^2^ = 0%) and nonabsorbable subgroups evaluated by physician (SMD, −0.08, 95% CI, −0.39 to 0.23, *P* = 0.61, 4 RCTs,^38^, ^41^, ^42^, ^44^ *I* ^2^ = 0%). The subgroup difference was not significant (*P* = 0.49). The results evaluated by the patients could not be assessed due to the lack of data in the absorbable suture subgroup.

Subgroup analysis by the CTA type could not be assessed due to the lack of data in the short‐chain CTA subgroup.

When subgroup analysis by age group was performed, there were no significant differences between groups evaluated by the physicians: adults (SMD, −0.22, 95% CI, −0.76 to 0.31, *P* = 0.42, *I* ^2^ = 0%, 2 RCTs^44^, ^46^) and children (MD, 0.17, 95% CI, −0.28 to 0.63, *P* = 0.46, 1 RCT^36^). The subgroup difference was not significant (*P* = 0.27). The results evaluated by the patients could not be assessed due to the lack of data in the children subgroup.

When subgroup analysis by types of wound was performed, there were no significant differences between surgical incision (SMD, −0.14, 95% CI, −0.44 to 0.16, *P* = 0.35, 4 RCTs,^38^, ^41^, ^44^, ^46^ *I* ^2^ = 0%) and laceration subgroups (MD, 0.17, 95% CI, −0.28 to 0.63, *P* = 0.46, 1 RCT^36^) evaluated by physician. The subgroup difference was not significant, *P* = 0.26. The results evaluated by the patients could not be assessed due to the lack of data in the laceration subgroup.

#### Scar Depth

The scar depth was measured from the wound edge to the deepest part of the wound. One study^40^ assessed the scar depth by the physicians at >1‐ to ≤3‐month period. The suture group had a significantly deeper scar than the CTA group (MD, 0.26, 95% CI, 0.13‐0.39, *P* < 0.0001, 1 RCT^40^).

#### Scar Width

One study^51^ analyzed scar width by the physicians at >3 months to 12 months. The width was measured at the midpoint and the widest point, the suture scars were significantly wider than those of the CTAs (MD, 0.67, 95% CI, 0.30‐1.04, *P* = 0.0003, 1 RCT^51^ and MD, 0.93, 95% CI, 0.45‐1.41, *P* = 0.0003, 1 RCT,^51^ respectively).

#### Pain

Two papers reported the data.^36^, ^47^ Pain intensity during the procedure was assessed in 1 study^36^ using the VAS, which had been validated and shown to be accurate in pain measurement.^53^, ^54^ The CTA group had significantly less pain (MD, −19.00, 95% CI, −33.14 to −4.86, *P* = 0.008, 1 RCT^36^). However, another paper^47^ presented data in nonparametric statistic.

#### Closure Time

**Figure 3:**
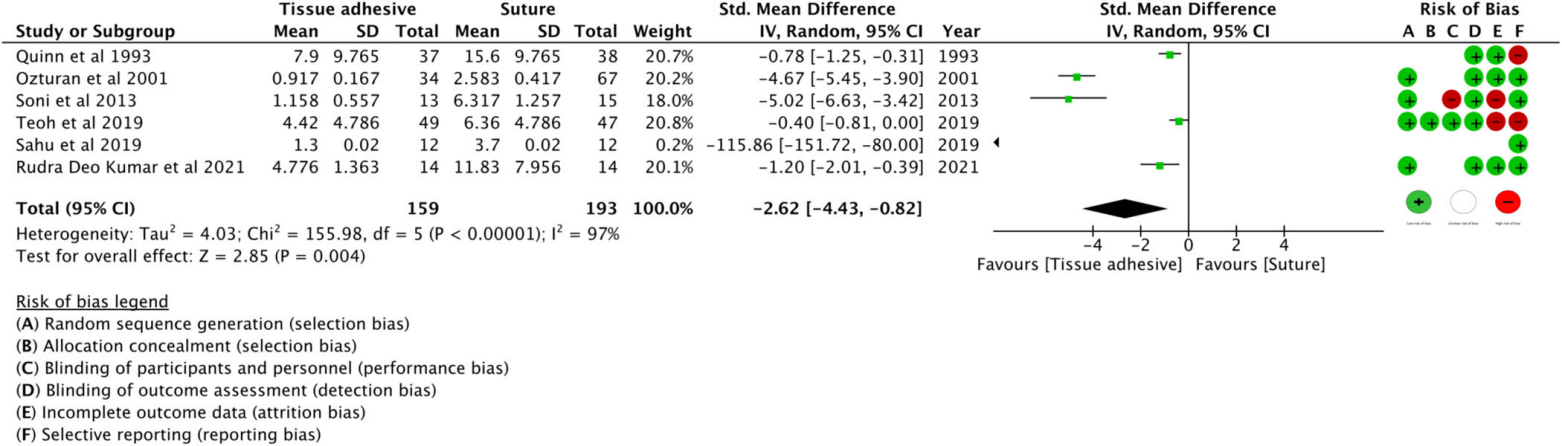
Closure time. CI, confidence interval; IV, inverse variance; Random, random effects; Std., standardized mean difference.

The closure time was recorded from when the skin was completely prepared to when the surgeon's hands were removed from the wound. Eight studies^36^, ^37^, ^38^, ^44^, ^48^, ^49^, ^50^, ^52^ reported the closure time. However, the SD of 1 study^37^ could not be imputed and another study^48^ reported the time of skin closure per centimeter of incision without any information on the incision length. Figure 3 shows that the closure time of the CTA group was less than that of the suture group (SMD, −2.62, 95% CI, −4.43 to −0.82, *P* =0.004, 6 RCTs,^36^, ^38^, ^44^, ^49^, ^50^, ^52^ *I* ^2^ = 97%).

Sensitivity analysis was performed by excluding 1 RCT^49^ with the highest extreme values. The analysis revealed that the CTA group had less closure time than the suture group (SMD, −2.32, 95% CI, −3.89 to −0.75, *P* = 0.004, 5 RCTs,^36^, ^38^, ^44^, ^50^, ^52^ *I* ^2^ = 97%).

#### Cost

The cost was mentioned in 2 articles.^38^, ^42^ The cost of material was higher in the adhesive group.^42^ However, the total closure cost (cost of material, cost of transportation for follow‐up, cost for loss of wages, cost of dressing, and local antimicrobial medication) was higher in the suture group.^38^, ^42^ This domain could not be analyzed in the meta‐synthesis, because the statistical data from the original studies could not be imputed.

#### Adverse Events

**Figure 4:**
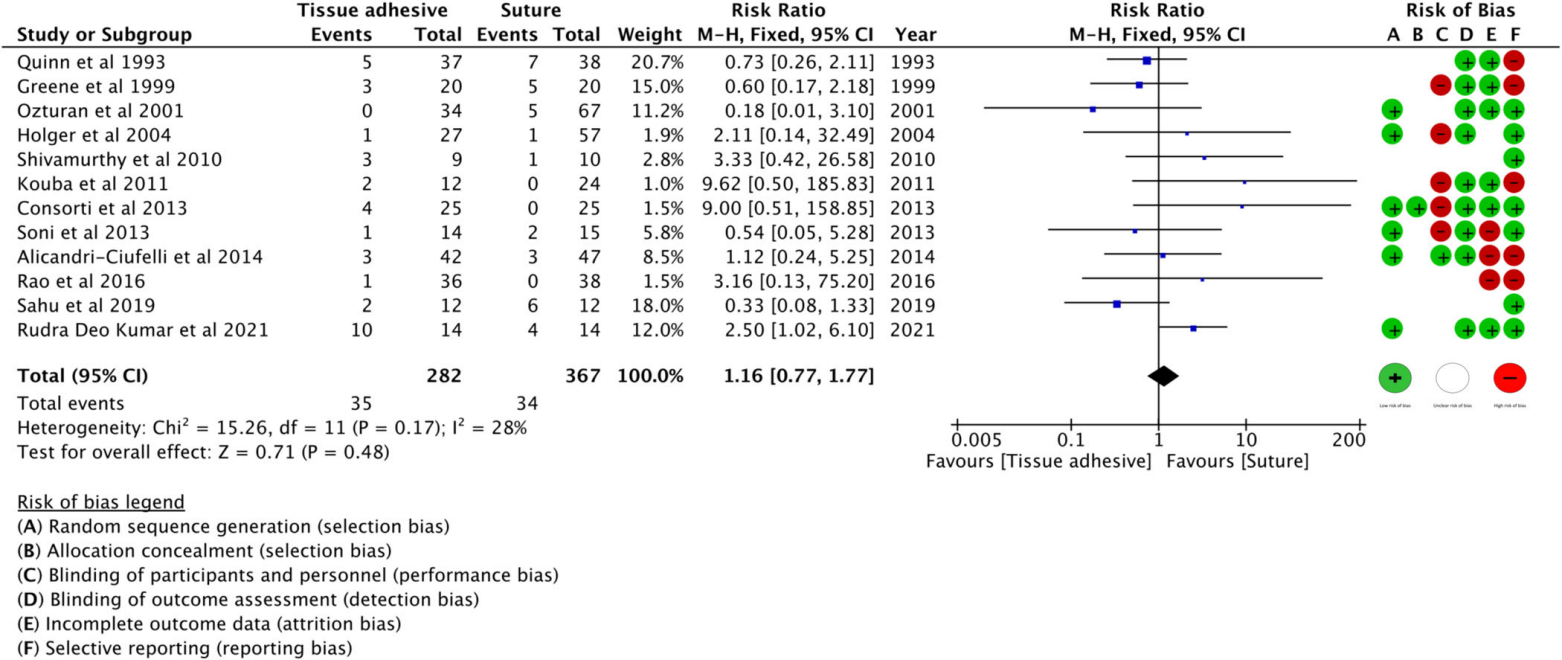
Overall adverse events. CI, confidence interval; Fixed, fixed effect; M‐H, Mantel‐Haenszel analysis.

Twelve studies^33^, ^36^, ^37^, ^38^, ^39^, ^42^, ^43^, ^44^, ^45^, ^47^, ^49^, ^52^ compared overall adverse events between the CTA and suture groups. Figure 4 shows no significant difference in the risk of adverse events between the 2 groups (RR: 1.16, 95% CI, 0.77‐1.77, *P* = 0.48, 12 RCTs,^33^, ^36^, ^37^, ^38^, ^39^, ^42^, ^43^, ^44^, ^45^, ^47^, ^49^, ^52^ *I* ^2^ = 28%). When common complications were assessed, there were no significant differences between groups in infection/inflammation (RR: 0.68, 95% CI, 0.28 to 1.65, *P* = 0.39, 8 RCTs,^36^, ^37^, ^38^, ^39^, ^42^, ^45^, ^49^, ^52^ *I* ^2^ = 0%), erythema (RR: 0.44, 95% CI, 0.12‐1.66, *P* = .23, 3 RCTs,^36^, ^44^, ^52^ *I* ^2^ = 0%), dehiscence (RR: 2.12, 95% CI, 0.99‐4.53, *P* = 0.05, 9 RCTs,^33^, ^36^, ^37^, ^39^, ^42^, ^44^, ^47^, ^49^, ^52^ *I* ^2^ = 0%), and bleeding (RR: 2.15, 95% CI, 0.66‐7.03, *P* = 0.21, 3 RCTs,^42^, ^43^, ^45^ *I* ^2^ = 18%).

#### Risk of Bias

**Figure 5:**
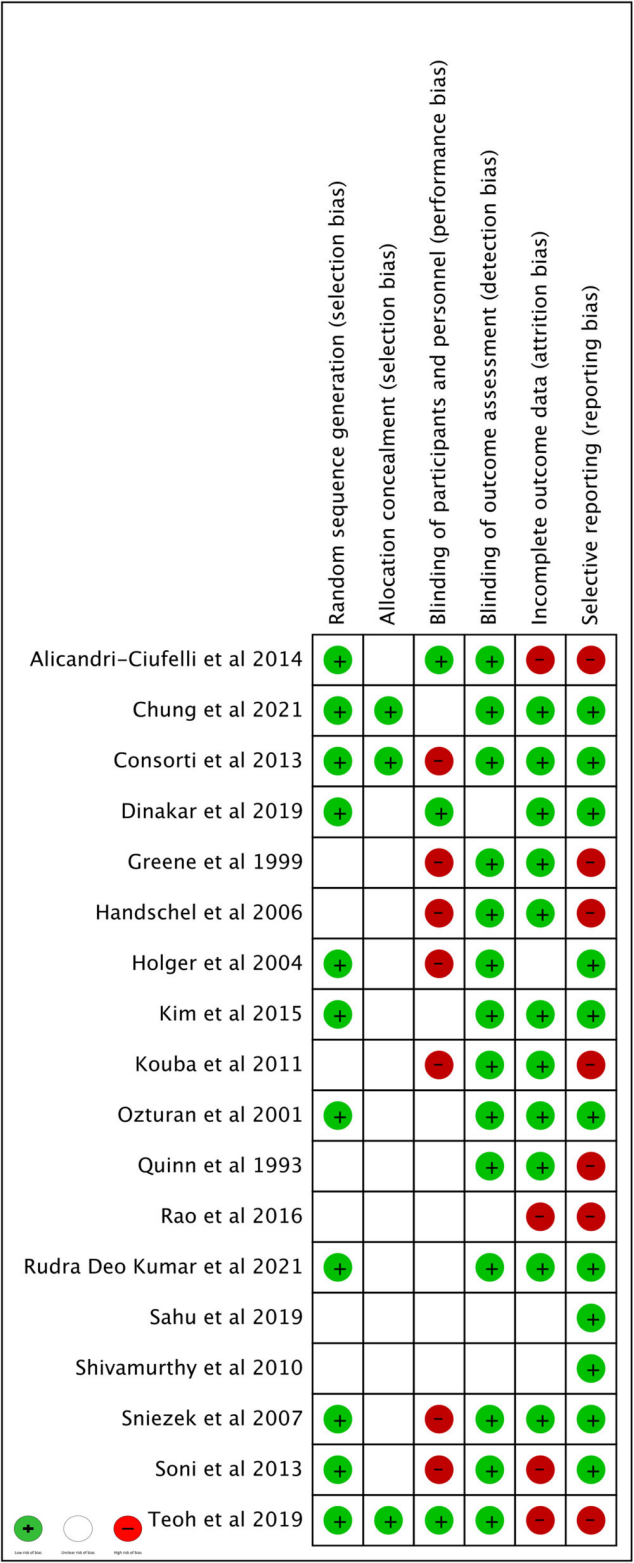
Risk of bias summary: Review authors' judgments about each risk of bias item for each included study.

The risk of bias assessment is presented in Figure 5. In summary, 11 trials (61.11%) were at low risk of bias in random sequence generation, incomplete outcome data, and selective reporting. Fourteen trials (77.78%) adequately generated blinding outcome assessments. Potential sources of bias resulted from allocation concealment and blinding participants and personnel with only 3 trials (17.65%) placed at low risk of bias in each outcome.

#### Publication Bias

**Figure 6:**
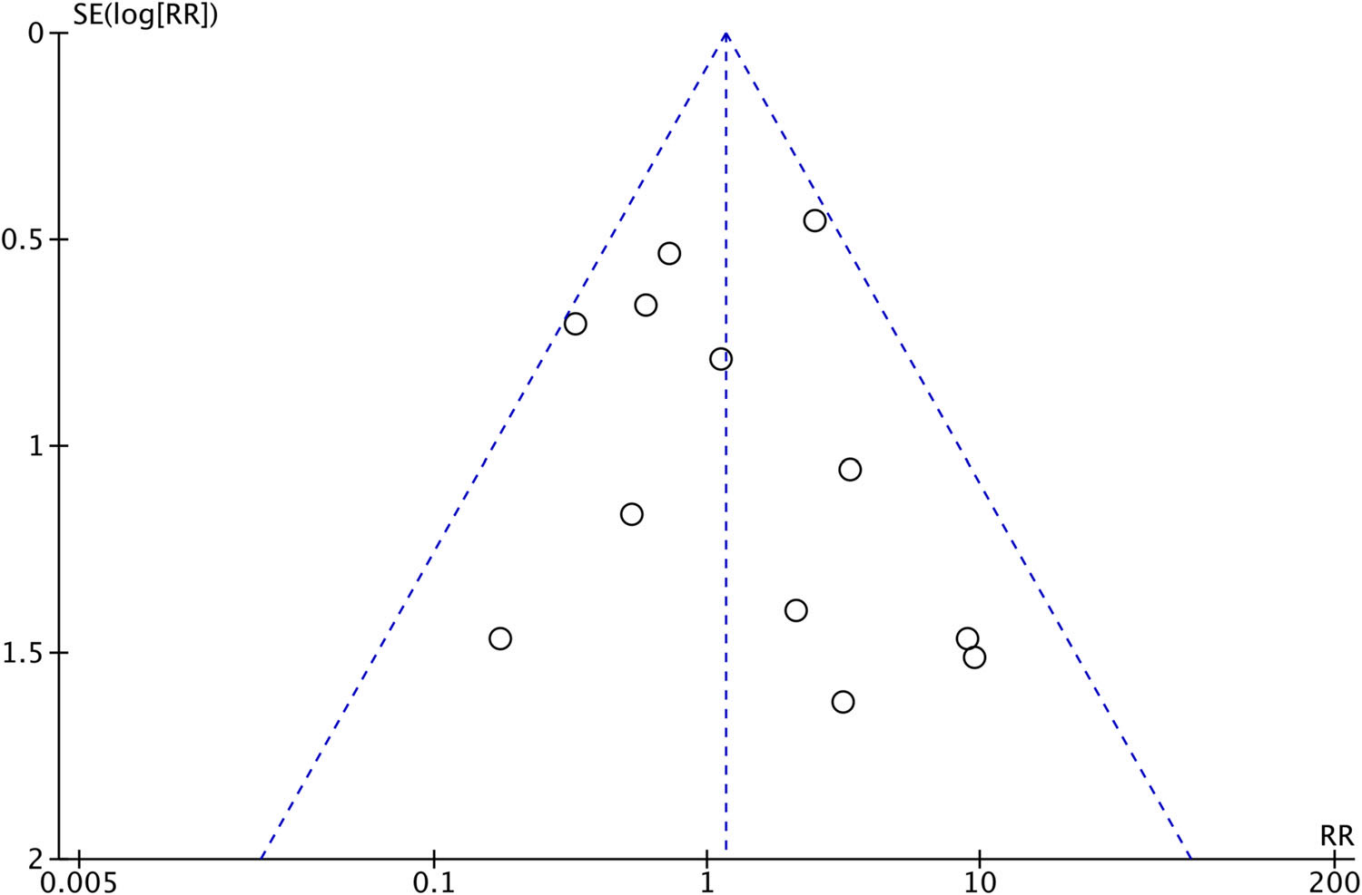
A funnel plot analyzing publication bias. RR, risk ratio; SE, standard error.

Publication bias was implemented to assess the potential exaggeration of the genuine effect size in instances where the number of incorporated studies exceeded 10.^23^ Studies demonstrating adverse events were used to draw a funnel plot to analyze publication bias. The selected articles^33,36‐39,42‐45,47,49,52^ were symmetrically distributed in a funnel plot (Figure 6).

## Discussion

The results from our systematic review and Meta‐analysis based on the wound healing process revealed that the cosmetic outcomes favored CTAs at ≤1 month. The scar quality was comparable between both groups. However, the Wound Registry Scale favored the CTAs group. This finding could be due to CTAs did not cause additional scars whereas sutures created more scars from stitches. Moreover, the post hoc analyses of the previous literature suggested that the more favorable outcomes of CTAs could be attributed to the CTA's ability to assist in elevating or everting the wound margins.^45^ At >1 to ≤3 months, the Meta‐analysis of cosmetic appearance evaluated by the VAS showed comparable scars. At >3 to 12 months, the cosmetic outcomes analyzed by the VAS were comparable. Nevertheless, the results analyzed by the POSAS favored sutures, this finding could be because of the suture material, STRATAFIX, which was used in the included study.^51^ STRATAFIX consists of spiral barbed sutures with anchors which can help grip tissue and improve the scar by reducing local tissue ischemia.^55^, ^56^ This reason may explain the result that favored the suture group. Therefore, it may be assumed that the cosmetic outcome would be comparable, except when the STRATAFIX was used as suture material.

The scar depth and width in the CTA group were less than those in the suture group at >1‐ to ≤3‐month and >3 to 12‐month assessments, respectively. The exact mechanism that results in better scar depth and width in the CTA group is still unknown. However, a theory is that CTAs may offer better oxygenation than sutures. The circumferential force on the wound from suture stitches could lead to local ischemia, especially if the suture stitches were too tight. Oxygenation is a local factor affecting wound healing and influences nearly all processes, including fibroblast proliferation and wound contraction mediated by myofibroblast, which can impact the scar size.^57^, ^58^, ^59^, ^60^, ^61^ Another possibility is that CTAs may help approximate the wound edges better than the sutures because the holding force in the wounds using CTAs is distributed along the wound edges, while the tension forces of sutures are at the stitch areas. Less tension can result in fewer scars.^57^, ^58^, ^59^, ^60^, ^61^ Nevertheless, the exact mechanism needs to be investigated in future studies. CTAs could provide additional benefits, including less pain, closure time, and total closure cost. Adverse events in the CTA group were similar to those of the suture group in any dimension.

There was only 1 RCT^36^ that evaluated pain in this Meta‐analysis. This RCT evaluated intraoperative pain under local anesthesia and favored the CTA group. However, postoperative or intraoperative pain under general anesthesia was not analyzed due to the limitation of the original data and, maybe, the feasibility of intraoperative pain assessment under general anesthesia.

A systematic review by Farion et al^21^ compared a CTA versus other standard wound closures (staple, suture, sterile strips) of linear, traumatic, and laceration wounds in all body areas. There were no differences between groups in cosmetic outcomes. However, the dehiscence rate slightly increased in the adhesive group. Likewise, Dumville et al^62^ demonstrated that the rate of wound dehiscence was significantly higher in the CTA group than of the suture group. However, both articles^21^, ^62^ evaluated mixed wound locations, some of which, such as the scalp or the extremities, may be highly mobile areas or areas with high tension. In contrast, our study selected only the low‐tension areas claimed to be suitable for using CTAs, according to the product properties. Our results showed that the rate of wound dehiscence was comparable. In the systematic reviews conducted by Raj et al,^63^ it was found that the long‐term cosmetic outcomes were similar. However, our study yielded a different result, indicating a better scar outcome on the suture side. This can be attributed to our inclusion of any type of suture material, including spiral barb sutures, in order to achieve a more desirable scar appearance.

From our study, adverse events were similar between the 2 groups. When subgroup analyses by wound locations, suture materials, age groups, and type of wounds were performed, there were no differences between the 2 groups, except in the neck subgroup the cosmetic appearance favored CTAs, but the statistical effects were minimal based on 1 RCT and were solely based on the patient viewpoint. This result needed to be further validated by additional studies. Specifically, factors related to scar outcomes of the included papers such as tension, infection, sterility did not show significant differences between the face and neck regions. However, the subgroup analysis by the CTA type could not be assessed due to a lack of data from the short‐chain CTAs. Further research assessing the effectiveness of short‐chain CTAs compared with sutures maybe needed. Although aging altered the wound healing capacity, its effects were primarily observed in chronic wounds.^64^ Unlike acute injuries, which were a characteristic of the wounds in our study, so no differences were detected among the age groups.

Based on the findings of the present study, CTAs can be used as an option for facial and neck wounds. The aesthetic outcomes of CTAs were favored than those of the suture group at ≤1 month and then the outcomes were comparable up to 3 months. Additionally, they could save time, total cost of closures, and decrease pain, as well as offer less scar depth and width. Nonetheless, sutures exhibited superior cosmetic outcomes compared to CTAs at >3 to 12 months due to the spiral barbed sutures. These findings show that CTAs can be used as an alternative method for wound closure on the face and neck in clinical practice in any age group. Though CTAs demonstrated satisfactory outcomes, there is a limitation that they could be used only in specific wound characteristics such as low‐tension, laceration, or surgical incision wounds.

To the best of our knowledge, this study represents the inaugural Meta‐analysis comparing pooled data between CTAs and sutures for facial and neck wound closures, while comprehensively assessing scar appearance over short‐ and long‐term periods. Notably, only RCTs were considered for analysis. However, this study encountered several limitations, including the utilization of various tools for evaluating cosmetic outcomes and variations in the time points used to assess these outcomes. These factors posed challenges to the Meta‐analysis. Nevertheless, we mitigated these issues by selecting only validated measurement tools for evaluating cosmetic outcomes and conducting separate analyses according to the measurement tool. Furthermore, time points for outcome assessment were segregated based on wound healing principles, resulting in low heterogeneity across most outcomes within this study. Additionally, subgroup analyses demonstrated similarly low levels of heterogeneity. Unfortunately, due to a lack of original quantitative data, interesting outcomes such as cost could only be presented in a qualitative synthesis. Finally, it is important to note that the number of studies incorporated to estimate cosmetic outcomes at different time points was limited, necessitating further studies to strengthen the conclusion.

## Conclusion

From this systematic review and Meta‐analysis, the assessment based on the wound healing process showed that the cosmetic appearance outcomes indicated a preference for CTAs at ≤1 month. The results were comparable at the > 1 to ≤3 months. However, sutures exhibited superior cosmetic outcomes compared to CTAs at >3 to 12 months due to the spiral barbed sutures. Subgroup analyses revealed no differences in the cosmetic outcomes. The CTAs offered less scar depth, scar width, and total closure cost, caused less pain, and shortened the closure time. Adverse events were similar between the 2 groups. Consequently, CTAs may be considered as an alternative for closing wounds on the face and neck.

## Author Contributions

**Prapitphan Charoenlux**, conceptualization, data curation, formal analysis, investigation, methodology, project administration, resource, software supervision, validation, visualization, writing—original draft, writing—review and editing; **Nattawan Utoomprurkporn**, conceptualization, data curation, methodology, validation, visualization, writing—review and editing; **Kachorn Seresirikachorn**, conceptualization, data curation, formal analysis, investigation, methodology, project administration, resource, software supervision, validation, visualization, writing—review and editing.

## Disclosures

### Competing interests

All authors have no conflict of interest to declare.

### Funding source

None.
